# Many neighbors are not silent. fMRI evidence for global lexical activity in visual word recognition

**DOI:** 10.3389/fnhum.2015.00423

**Published:** 2015-07-22

**Authors:** Mario Braun, Arthur M. Jacobs, Fabio Richlan, Stefan Hawelka, Florian Hutzler, Martin Kronbichler

**Affiliations:** ^1^Neurocognition Lab, Centre for Cognitive Neuroscience, Universität SalzburgSalzburg, Austria; ^2^Department of Experimental and Neurocognitive Psychology, Freie Universität BerlinBerlin, Germany; ^3^Center for Cognitive Neuroscience BerlinBerlin, Germany; ^4^Dahlem Institute for Neuroimaging of Emotion, BerlinGermany; ^5^Christian-Doppler-Klinik, Paracelsus Medical University, SalzburgAustria

**Keywords:** visual word recognition, neighborhood density effect, mental lexicon, orthographic similarity, dorso- and ventromedial cortex, fast-guess mechanism, deadline mechanism, identification mechanism

## Abstract

Many neurocognitive studies investigated the neural correlates of visual word recognition, some of which manipulated the orthographic neighborhood density of words and nonwords believed to influence the activation of orthographically similar representations in a hypothetical mental lexicon. Previous neuroimaging research failed to find evidence for such global lexical activity associated with neighborhood density. Rather, effects were interpreted to reflect semantic or domain general processing. The present fMRI study revealed effects of lexicality, orthographic neighborhood density and a lexicality by orthographic neighborhood density interaction in a silent reading task. For the first time we found greater activity for words and nonwords with a high number of neighbors. We propose that this activity in the dorsomedial prefrontal cortex reflects activation of orthographically similar codes in verbal working memory thus providing evidence for global lexical activity as the basis of the neighborhood density effect. The interaction of lexicality by neighborhood density in the ventromedial prefrontal cortex showed lower activity in response to words with a high number compared to nonwords with a high number of neighbors. In the light of these results the facilitatory effect for words and inhibitory effect for nonwords with many neighbors observed in previous studies can be understood as being due to the operation of a fast-guess mechanism for words and a temporal deadline mechanism for nonwords as predicted by models of visual word recognition. Furthermore, we propose that the lexicality effect with higher activity for words compared to nonwords in inferior parietal and middle temporal cortex reflects the operation of an identification mechanism based on local lexico-semantic activity.

## Introduction

Successful visual word recognition involves the synchronized interplay of multiple sensory-motor, attentional and memory networks. Classical neurological models and current neuroimaging results suggest a set of left hemispheric regions comprising the inferior temporal, inferior frontal, supramarginal, and angular gyri to be strongly involved in this process (Geschwind, 1965; Damasio and Geschwind, 1984; Bookheimer, 2002; Binder et al., 2005; Price, 2012). On the stimulus side, a vast number of sublexical and lexical variables have been shown to influence word recognition (e.g., bi- or trigram, syllable, and word frequency) in a wide variety of tasks (e.g., perceptual identification, lexical, or semantic decision, naming, silent reading). Among the over 50 quantifiable factors known to affect word recognition performance (Graf et al., 2005), one of the most prominent variables is orthographic neighborhood density, i.e., the number of orthographic neighbors, which can be generated by changing one letter of a given word (Coltheart et al., 1977). When subjects make lexical decisions to words and nonwords, a standard finding is that responses to words with high neighborhood density are faster compared to words with low neighborhood density (Andrews, 1989; Jacobs and Grainger, 1992, 1994; Sears et al., 1995; Forster and Shen, 1996; Grainger and Jacobs, 1996; Carreiras et al., 1997; see Hawelka et al., 2013 for effects in natural reading). On the other hand, response times to nonwords show a reversed effect: responses are slower for nonwords with high compared to those with a low number of neighbors. The effect is of interest because it is assumed to reflect a direct top–down influence of memory representations on the perception of a letter string which has played a significant role in the development of computational models of word recognition and reading (Jacobs and Grainger, 1994). One explanation for the observed lexicality by neighborhood interaction is that during the early stages of visual word recognition a letter string activates orthographically similar word representations in a hypothetical mental lexicon. In the case of stimuli with a high number of neighbors this is assumed to result in the activation of a larger number of candidate representations compared to those with a low number of neighbors. In interactive activation and hybrid dual-route models of visual word recognition (McClelland and Rumelhart, 1981; Grainger and Jacobs, 1996; Coltheart et al., 2001; Hofmann et al., 2011; Hofmann and Jacobs, 2014) this activation can directly be computed on the basis of the summed activity over all lexical units, i.e., the amount of global lexical activity for both words and nonword stimuli thus providing a quantitative predictor for both behavioral and neurocognitive studies of word recognition. Jacobs and Carr (1995) speculated that levels of neural activity in the left medial prestriate cortex vary systematically with the levels of computational activity predicted to occur in the orthographic lexicon of interactive activation models. While this speculation was never directly tested, there is some neurocognitive evidence indicating that Jacobs and Carr’s idea of a cross-fertilization between computational modelers and mind mappers in the domain of reading was not too far-fetched. Until now, however, neurocognitive evidence for the organization and possibly distributed locations of such a neural correlate of a hypothetical mental lexicon activated by words and nonwords is still scarce.

Some evidence for global lexical activity as the basis of neigbhorhood density effects was found in electrophysiological research (Holcomb et al., 2002; Braun et al., 2006). Holcomb et al. (2002) observed greater N400 effects for words and nonwords with a high number of neighbors compared to those with a low number of neighbors in lexical decision, as well as a greater N400 and N150/350 in a go/no-go semantic categorization task, but did not directly relate these findings to output from a computational model. Later, Braun et al. (2006) did exactly this by using ERPs to test the hypothesis of a global activation of representations of orthographically similar words. Two mechanisms implemented in the multiple read out model of visual word recognition (MROM; Jacobs and Grainger, 1994; Grainger and Jacobs, 1996; Jacobs et al., 1998) were proposed to be in effect in lexical decisions to words and nonwords in their study: first, an early identification mechanism for stored representations of words around 300 ms supposed to reflect local, i.e., word specific, lexical activity and to underly ‘yes’ responses to words; second, a temporal deadline mechanism around 500 ms assumed to reflect global, i.e., non-specific, lexical activity in a hypothetical mental lexicon and to underly ‘no’ responses to nonwords (see also Barber and Kutas, 2007).

Neuroimaging research using neighborhood density as a measure of orthographic similarity so far provided only little evidence for higher activity in response to words or nonwords with a high number of neighbors (Binder et al., 2003; Fiebach et al., 2007). Rather, blood oxygen level dependent (BOLD) responses were observed to be higher for stimuli with a low number of neighbors. Binder et al. (2003) found higher activity in response to words without neighbors in left prefrontal, angular gyrus, and ventrolateral temporal areas which was interpreted to reflect the fact that accurate responses in lexical decisions depend on the activation of semantic information. Thus, although not being directly comparable, the results of Binder et al. (2003) are somewhat at odds with the brain-electrical findings of greater lexico-semantic effects for items with a high number of neighbors relative to those with a low number of neighbors (Holcomb et al., 2002; Braun et al., 2006).

A second fMRI study (Fiebach et al., 2007) reported greater activation for stimuli with a low number of neighbors in the superior temporal sulcus and the angular gyrus (although this main effect of neighborhood density did not exceed the significance threshold) thus replicating in part the results of Binder et al. (2003). In addition, the analysis showed a lexicality by neighborhood density interaction in the left mid-dorsolateral prefrontal cortex, more specifically in the posterior inferior frontal sulcus and middle frontal gyrus, and in a region slightly anterior to the pre-SMA in the medial superior frontal gyrus. Activity in the mid-dorsolateral prefrontal cortex was strongest in response to nonwords with a high number of neighbors. In contrast, activity in the medial superior frontal gyrus was stronger for words with a low number of neighbors. Fiebach et al. (2007) interpreted this activity in frontal regions to reflect domain-general processing at a late post-lexical level rather than reflecting activity associated with a hypothetical mental lexicon.

Although, the metaphor of a ‘mental lexicon’ storing the visual form of words which are co-activated when similar items are presented is part and parcel of almost all current computational models of word recognition (regardless of whether they use localist or distributed units; cf. Jacobs and Grainger, 1994), the neurocognitive literature dealing with this notion is still inconclusive and the model-to-brain-data connection is still weak, despite some recent progress (e.g., Levy et al., 2009; Taylor et al., 2012; Hofmann and Jacobs, 2014). A likely candidate for a hypothetical mental lexicon is Wernicke’s area in left posterior superior and middle temporal lobe since this region was repeatedly found to be involved in language comprehension (e.g., Howard et al., 1992; Beauregard et al., 1997). Many studies report that the left middle temporal gyrus is consistently more active during the processing of words than during the processing of nonwords (e.g., Hagoort et al., 1999; Fiebach et al., 2002; Binder et al., 2003). Activation in the middle temporal gyrus is suggested to signal either semantic processing (e.g., Price et al., 1997; Indefrey and Levelt, 2004; Gold et al., 2005; Cao et al., 2006; Vigneau et al., 2006; Booth et al., 2007; Richlan et al., 2009; Newman and Joanisse, 2011; Whitney, 2011; Noonan et al., 2013), or phonological processing (e.g., Indefrey and Levelt, 2004; Bitan et al., 2005; Brambati et al., 2009; Simos et al., 2009; Newman and Joanisse, 2011; Graves et al., 2014), or orthographic-phonological mapping (e.g., Graves et al., 2010).

Furthermore, neurological evidence from Wernicke aphasics shows that these are unable to semantically categorize words (e.g., Zurif et al., 1974), or to explicitly judge words on the basis of semantic information (Goodglass and Baker, 1976), leading to the conclusion that controlled lexico-semantic processes are deficient in these patients (Milberg et al., 1987; see Friederici, 1998 for a review). Thus, previous research suggests that the superior and middle temporal gyri are likely regions for hosting a hypothetical mental lexicon despite the lack of evidence for higher global lexical activity for words or nonwords.

Another prominent brain region for being part of a hypothetical mental lexicon is the ventral occipitotemporal region, hosting the visual word form area (VWFA; Cohen et al., 2000b). A great deal of research showed that the ventral occipitotemporal region is involved in the identification of letters and words (e.g., Cohen et al., 2000b, 2004, 2008; Vinckier et al., 2007). Vinckier et al. (2007) observed a posterior to anterior specialization within the ventral occipitotemporal cortex with the anterior part showing the highest activity in response to words or word like stimuli.

The VWFA is thought to be important for the prelexical identification of letters and letter combinations. Research showed that VWFA activity associated with the identification of letters is independent of size, case, location or font (Dehaene et al., 2005), suggesting the computation of perceptually higher-order invariant orthographic units from the input. This information is then thought to be transmitted to other regions involved in visual word recognition, such as the temporal, parietal, and inferior frontal regions (Cohen et al., 2004) which allow for further phonological and semantic processing.

Beside this proposed prelexical function other findings suggest a possible role for the VWFA in lexical processing (e.g., Cohen et al., 2004; Kronbichler et al., 2004, 2007; Bruno et al., 2008; Hauk et al., 2008; Glezer et al., 2009; Schurz et al., 2010; Baeck et al., 2015). For example, Kronbichler et al. (2007) reported activation differences in the VWFA by comparing words, pseudowords and pseudohomophones in a visual phonological decision task. Words elicited less activity compared to pseudohomophones and pseudowords which did not differ in activity. Their explanation for this finding was that visually presented words match onto stored representations leading to less activity compared to visually presented pseudowords which do not. Therefore, Kronbichler et al. (2007) suggested that this region not only computes letter string representations, but could be a region which also stores word specific orthographic information (i.e., orthographic lexicon function). A third function of the ventral occipitotemporal region in addition to the prelexical and lexical ones was suggested by Devlin et al. (2006) who proposed that the VWFA acts as a general interface area between bottom–up sensory information from different modalities and top–down higher order conceptual information. Word recognition is assumed to involve reciprocal interactions between sensory cortices and higher order processing regions via a hierarchy of forward and backward connections with sensory areas sending bottom–up information and higher-order regions sending top–down predictions which are based on prior experience and serve to resolve uncertainty about the sensory input (Dehaene and Cohen, 2011; Price and Devlin, 2011; see also Schurz et al., 2014a).

The present study was designed to further investigate the neural basis of the neighborhood density effect which provides important information about the structure and functioning of mental representations of words, i.e., the hypothetical mental lexicon, as conceptualized in extant computational models of word recognition (e.g., Grainger and Jacobs, 1996; Coltheart et al., 2001; Perry et al., 2007; Hofmann and Jacobs, 2014).

Finding differences in brain activity in response to words and nonwords with high or low numbers of neighbors in ventral occipitotemporal, inferior parietal, and/or middle temporal cortex would support the orthographic similarity/global lexical activity account as the basis of the neighborhood density effect and thus strengthen the above computational models. In contrast, activation in prefrontal cortex could suggest an extra-lexical locus of the effect and would thus provide no neuroimaging evidence for the existence and location of a mental lexicon as proposed by the models (Fiebach et al., 2007).

We employed a silent reading paradigm in the scanner to avoid potential confounds with executive task demands like decision and response related processes. Previous studies (Holcomb et al., 2002; Binder et al., 2003; Braun et al., 2006) mostly used the lexical decision task to investigate activation elicited by items with high and low number of neighbors which makes it difficult to distinguish between extra-lexical and lexical processes (Fiebach et al., 2007). Furthermore, we controlled the words and nonwords on a number of sublexical and lexical measures known to influence visual word processing (see **Table 1**) and used only short words (four letter in length) posing only low demands on the reading process itself.

**Table 1:** Means and (SD) for controlled variables for words and nonwords.

	Words		Nonwords	
	Low	High	Low	High
L	4	4	4	4
N	2(2)	6(2)	2(1)	6(2)
F	124(328)	97(164)	–	–
FN	1623(4628)	2423(6105)	894(1753)	2230(5119)
BiF	2247(4160)	3945(8085)	2655(5089)	4079(6660)

*L, Number of letters, N, number of orthographic neighbors, F, word frequency per million, FN, summed frequency of neighbors, BiF, summed bigram frequency count.*

## Materials and Methods

### Ethics

The study was approved by the ethics committee of the University of Salzburg (“Ethikkommission der Universität Salzburg”) and was in accordance with the principles expressed in the declaration of Helsinki. Informed consent was obtained from all participants.

### Participants

Twenty-two healthy participants (15 women) participated in the fMRI experiment. All were right-handed native German speakers and had no history of neurological disorders and normal or corrected to normal vision. Age ranged from 18 to 44 years. Two subjects were excluded from the analysis because of a problem with stimulus presentation. Subjects were recruited by students of the University of Salzburg, received course credit and were offered a CD with their anatomical fMRI scans. Subjects were tested individually at the Centre for Cognitive Neuroscience at the University of Salzburg.

### Experimental Materials and Procedure

Brain activity responses to 100 monosyllabic words and 100 nonwords were collected in two sessions together with two other experiments with 600 stimuli in total. The 200 stimuli were all four letters in length. The nonwords were pronounceable according to German pronunciation rules. The 200 stimuli were split into four groups to investigate the neighborhood density effect. Of the 100 words 50 had a low number of neighbors (three or less than three) the other 50 had a high number of neighbors (four or more). The same manipulation was applied to the 100 nonwords. The stimuli were matched between conditions for number of letters (let), word frequency (F), summed bigram frequency (BiF), and summed frequency of the neighbors (FN; see **Table 1**). Frequency counts were taken from the CELEX lexical database (Baayen et al., 1993).

Subjects were asked to read the stimuli (words/nonwords) silently while being in the scanner. To make sure the task was clear, subjects performed a practice session with 20 items on a laptop outside the scanner. The scanning session took about 50 min and the whole experiment took about 1 h and 30 min. There were 5 min of anatomical scanning, followed by two sessions of 21 min actual testing with a short break in between to ensure that the subjects felt comfortable and could remain concentrated. Stimuli were presented on a 1024 × 768 pixel screen in white font on black surface projected on a mirror inside the scanner. The font used was “Arial” with 50 pt size. Words and nonwords were presented in random order for 700 ms, after each stimulus a blank screen with a fixation cross was presented. Presentation times of the fixation cross were jittered: 166 fixation crosses were presented for 2500 ms, 20 for 3200 ms, eight for 7500 ms, and six for 10500 ms. The experimental software used was Presentation software from Neurobehavioral Systems^1^ (San Francisco, CA, USA). On an irregular basis a four to seven letter male or female name (10 in total) was presented. Subjects were instructed to respond by pressing a button with the index finger of their right hand of a MRI compatible button box whenever a name was presented during testing. This test was administered to ensure subjects attentive reading of all stimuli.

### Image Acquisition

Functional and structural imaging was performed with a Siemens Tim Trio 3 Tesla using a 32-channel head coil (Siemens, Erlangen, Germany). A gradient echo field map (TR 488 ms, TE 1 = 4.49 ms, TE 2 = 6.95 ms) and a high resolution (1 mm× 1 mm× 1.2 mm) structural scan with a T1 weighted MPRAGE sequence were acquired from each participant. The structural images were followed by two runs with 510 volumes each of functional images sensitive to BOLD contrast acquired with a T2^∗^ weighted gradient echo EPI sequence (TR = 2520ms, TE = 33 ms, flip angle = 77°, number of slices = 36, slice thickness = 3 mm, 64 × 64 matrix, FOV = 192 mm). Six dummy scans were acquired at the beginning of each functional run before stimulus presentation. Low frequency noise was removed with a high-pass filter (128 s).

For preprocessing and statistical analysis, SPM8 software^2^, running in a MATLAB 7.6 environment (Mathworks Inc., Natick, MA, USA), was used. Functional images were realigned, unwarped, corrected for geometric distortions using the fieldmap of each participant and slice time corrected. The high resolution structural T1 weighted image of each participant was processed and normalized with the VBM8 toolbox^3^ using default settings. Each structural image was segmented into gray matter, white matter and CSF and denoised and warped into MNI space by registering it to the DARTEL template provided by the VBM8 toolbox via the high-dimensional DARTEL (Ashburner, 2007) registration algorithm. Based on these steps, a skull stripped version of each image in native space was created. To normalize functional images into MNI space, the functional images were co-registered to the skull stripped structural image and the parameters from the DARTEL registration were used to warp the functional images, which were resampled to 3 mm × 3 mm × 3 mm voxels and smoothed with a 8-mm FWHM Gaussian kernel.

### fMRI Analysis

Statistical analysis was performed with a GLM two staged mixed effects approach. In the subject-specific first level model, each condition was modeled by convolving stick functions at its onsets with SPM8’s canonical hemodynamic response function and no time derivatives. On the subject-specific first level model conditions of interest were contrasted against the fixation baseline. These subject-specific contrast images were used for the 2nd level group analysis. Direct contrasts between words and nonwords with a high and low number of neighbors were calculated with 2 × 2 repeated measures ANOVAs and in case of a significant interaction with subsequent paired *t*-tests. For all statistical comparisons an uncorrected cluster threshold of *p* < 0.001 and a cluster extent of 25 was used. We decided to use a lenient threshold which is not uncommon in reading research (e.g., Martin et al., 2015, **Table 1**) to be able to find expected differences in a silent reading task which is known to elicit less brain activity compared to tasks which impose decision and/or manual responses (e.g., Fiebach et al., 2007). By setting the cluster extent to 25 we still allow for a correction of multiple comparisons according to the theory of Gaussian random fields (Kiebel et al., 1999). All stereotaxic coordinates for voxels with maximal *z*-values within activation clusters are reported in the MNI coordinate system.

## Results

### Imaging Results

#### Effect of Lexicality

**FIGURE 1:**
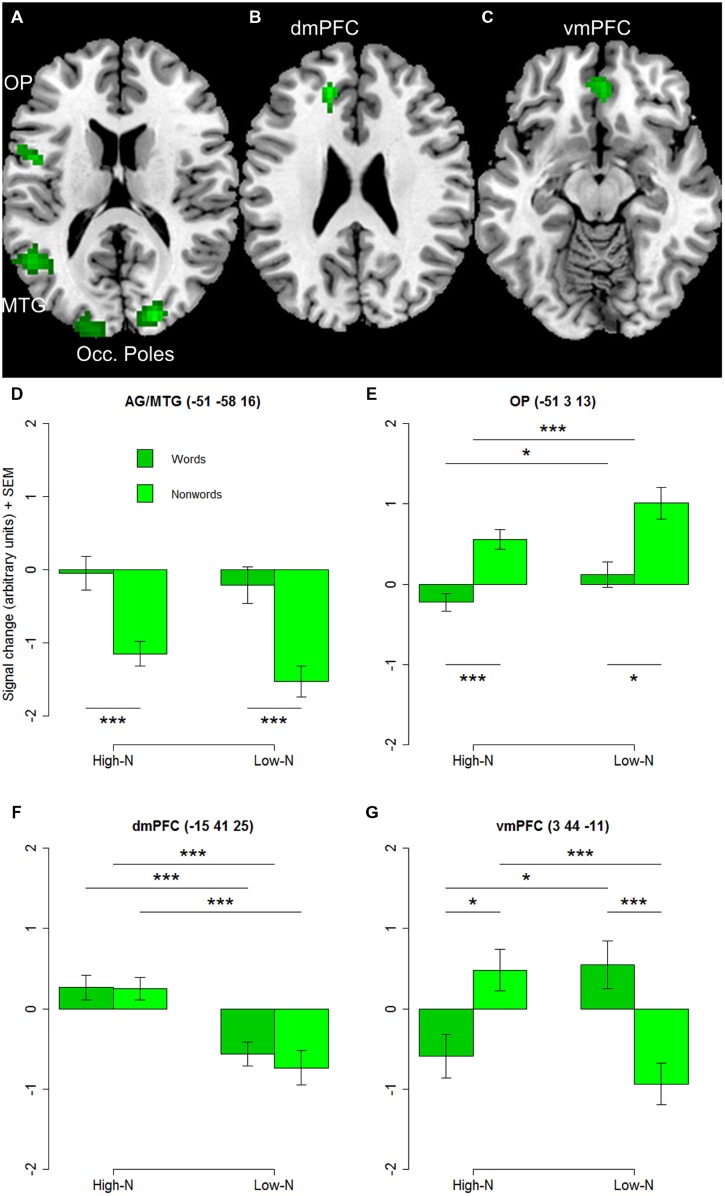
**(A)** Effect of lexicality showing greater activation for words compared to nonwords irrespective of neighborhood density in left angular and middle temporal gyrus (AG/MTG) and bilateral occipital poles. **(B)** Effect of neighborhood density with greater activation for words and nonwords with high number of neighbors compared to words and nonwords with low number of neighbors in dorsomedial prefrontal cortex (dmPFC). **(C)** Interaction of lexicality and neighborhood density with greater activity for words with low and nonwords with high number of neighbors in ventromedial prefrontal cortex (vmPFC). **(D–G)** Signal change for words and nonwords with high and low number of neighbors in AG/MTG, OP, dmPFC, vmPFC. AG, angular gyrus, MTG, middle temporal gyrus, OP, opercular cortex, Occ. Poles, occipital poles, dmPFC, dorsomedial prefrontal cortex, vmPFC, ventromedial prefrontal cortex. High-N, high number of neighbors, Low-N, low number of neighbors, SEM, Standard Error of the Mean. The whole-brain analysis was thresholded at *p* < 0.001, voxel level uncorrected and a cluster extent of 25. The displayed statistical differences are based on a 2 × 2 repeated measures ANOVA with Lexicality and Neighborhood density as within-subject factors for the respective region. Differences between levels of conditions show the results of paired *t*-tests with ^∗^*p* < 0.05 and ^∗∗∗^*p* < 0.005.

The whole-brain analysis showed effects of lexicality and neighborhood density as well as interactions between both factors. The lexicality effect was evident at bilateral occipital poles, the inferior parietal and middle temporal gyrus (**Figures 1A,D**; **Table 2**) with higher activity for words compared to nonwords. Furthermore, higher activity for nonwords compared to words was obtained in the precentral gyrus and opercular cortex (see **Figures 1A,E**; **Table 2**).

**Table 2:** Brain regions showing effects of lexicality and neighborhood density (voxel-level uncorrected, *p* < 0.001, cluster size > 25).

Brain region	Brodmann Area	Hemisphere	*x*	*y*	*z*	Cluster size	Zmax
**Effect of lexicality (words >nonwords)**	
Angular gyrus, lateral occipital cortex, superior division, middle temporal gyrus, temporooccipital part	21/37/39	L	-51	-58	16	77	4.26
Occipital Pole, lateral occipital cortex, superior division	18	R	21	-91	16	91	4.76
Occipital Pole	17	L	-15	-100	10	88	4.45
**Effect of lexicality (nonwords > words)**	
Precentral gyrus, opercular cortex, pars opercularis	6/48	L	-51	2	13	41	4.37
**Neighborhood density effect (high > low)**	
Dorsomedial prefrontal cortex (dmPFC), paracingulate gyrus	32	L	-15	41	25	29	4.16
**Lexicality × neighborhood density interaction**	
Ventromedial prefrontal cortex, paracingulate gyrus	11	L	3	44	-11	34	4.26

*x, y, z, peak coordinates according to MNI stereotactic space, cluster size in voxels; high, high number of neighbors, low, low number of neighbors.*

The separately performed 2 × 2 repeated measures ANOVAs for these regions with either higher activity for words compared to nonwords or vice versa with the beta estimates of the peak values with lexicality (words, nonwords) and neighborhood density (high, low) as within-subject factors showed main effects of lexicality in left AG/MTG: *F*(1,19) = 23.44, *p* < 0.001 and left precentral/opercular cortex: *F*(1,19) = 14.26, *p* = 0.001 and also a main effect of neighborhood density in the opercular cortex *F*(1,19) = 13.28, *p* = 0.002, and no interaction.

#### Effect of Neighborhood Density

The contrast of greater activity of high compared to low neighborhood density revealed significant differences in the dorsomedial prefrontal and left opercular cortex (see **Figures 1B,E,F**; **Table 2**). The 2 × 2 repeated measures ANOVA with lexicality (words, nonwords) and neighborhood density (high, low) as within-subject factors with the beta estimates of the peak values of high vs. low neighborhood density words and nonwords showed no main effect of lexicality [*F*(1,19) = 0.21, *p* = 0.655] and no interaction [*F*(1,19) = 0.19, *p* = 0.665, but a main effect of neighborhood density *F*(1,19) = 21.24, *p* < 0.001]. In contrast, no region showed greater activity for low compared to high neighborhood density words and nonwords at the chosen threshold (*p* < 0.001 uncorrected, cluster extent 25).

#### Lexicality by Neighborhood Density Interaction

Furthermore, the whole-brain analysis revealed an interaction of lexicality by neighborhood density in the ventromedial prefrontal cortex: words with a high number of neighbors showed lower activity compared to words with a low number of neighbors. The pattern of activity was reversed for the nonwords (see **Figures 1C,G**; **Table 2**). The 2 × 2 repeated measures ANOVA with lexicality (words, nonwords) and neighborhood density (high, low) as within-subject factors with the beta estimates of the peak values showed no main effect of lexicality [*F*(1,19) = 0.58, *p* = 0.457] and no main effect of neighborhood density [*F*(1,19) = 0.24, *p* = 0.63, but an interaction *F*(1,19) = 11.71, *p* = 0.003]. Paired *t*-tests showed that both words: *t* = -2.26, df = 19, *p* = 0.035, and nonwords: *t* = 3.25, df = 19, *p* = 0.004, showed an effect of neighborhood density (see **Figure 1G**).

#### ROI Analysis for Selected Regions Showing a Lexicality Effect

**FIGURE 2:**
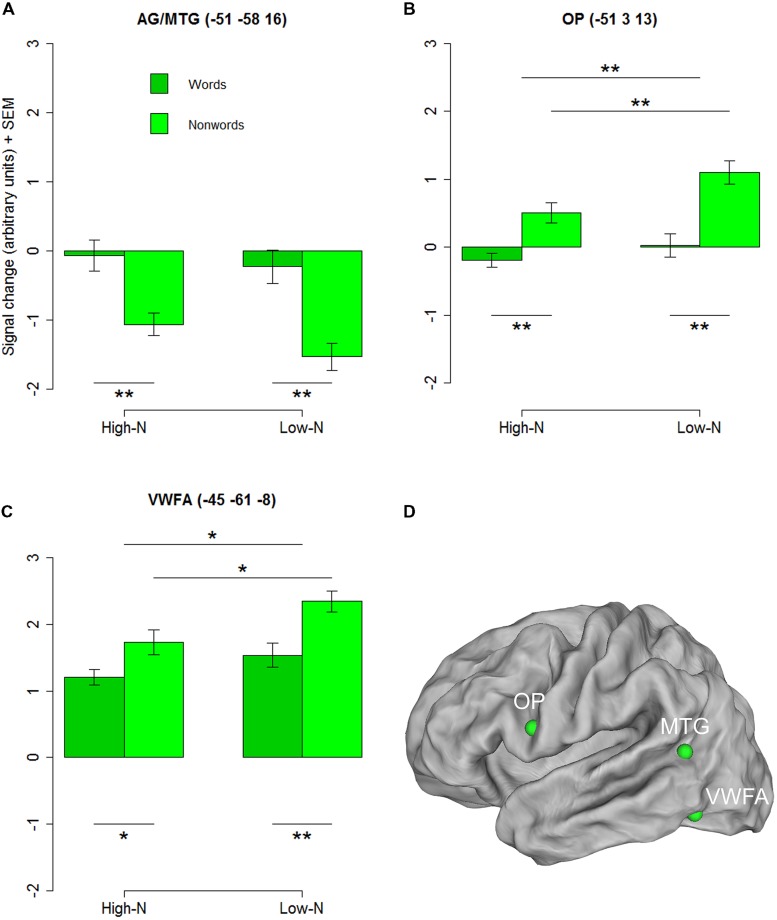
**Regions chosen for the additional ROI analysis showing an effect of lexicality in the whole-brain analysis.** AG/MTG **(A)** and opercular cortex **(B)** showed signficant effects at *p* < 0.001 (voxel level uncorrected, cluster extent 25). Surface representations for visualizing the ROIs in **(D)** were created using Caret software (Van Essen et al., 2001, http://brainmap.wustl.edu/caret/). Activity in the VWFA **(C)** did not pass the statistical cluster size criterion (25), which is not surprising because of the known inter-subject variability in the location of the VWFA (e.g., Glezer and Riesenhuber, 2013), but was chosen because of its suggested importance in visual word recognition. AG, angular gyrus, MTG, middle temporal gyrus, OP, opercular cortex; VWFA, visual word form area. High-N, high number of neighbors, Low-N, low number of neighbors, SEM, Standard Error of the Mean. The whole-brain analysis was thresholded at *p* < 0.001, voxel level uncorrected and a cluster extent of 25. The displayed statistical differences are based on a 2 × 2 repeated measures ANOVA with Lexicality and Neighborhood density as within-subject factors for the respective ROI. Differences between levels of conditions show the results of paired *t*-tests with ^∗^*p* < 0.05 and ^∗∗^*p* < 0.01.

To further investigate the basis of the neighborhood density effect and it’s relation to reading related areas we extracted three regions of interests (ROIs) identified by the contrasts of words vs. nonwords at the whole-brain level. Three ROIs were created by drawing 4-mm spheres around the peak coordinates in the opercular (-51 3 13) and the inferior parietal/middle temporal cortex (-51 -58 16) and in the inferior temporal gyrus near the location of the VWFA (-45 -61 -8). The 2 × 2 repeated measures ANOVAs with lexicality (lex; words/nonwords) and neighborhood density (n; high/low) as within subject factors for the three ROIs revealed a main effect of lexicality for the inferior parietal/middle temporal ROI [*F*(1,19) = 21.90, *p* < 0.001] and main effects of lexicality and neighborhood density for the opercular cortex [Flex(1,19) = 15.63, *p* = 0.002; Fn(1,19) = 12.86, *p* = 0.002] and the VWFA [Flex(1,19) = 8.83, *p* = 0.005; Fn(1,19) = 4.42, *p* = 0.029] ROIs and no interactions confirming the results of the whole-brain analysis (see **Figures 2A–D**).

## Discussion

Computational models of visual word recognition predict that words and nonwords with a high neighborhood density elicit high values of global lexical activity by activating orthographically similar entries in a hypothetical mental lexicon and that this activity is the basis for the facilitatory effects for words with many neighbors and the inhibitory effects for nonwords in lexical decision (e.g., Grainger and Jacobs, 1996; Coltheart et al., 2001; Braun et al., 2006). Simulations using these models show that high levels of global lexical activity lead to word present signals for both high-density words and nonwords, thus increasing both hit and false alarm rates in data-limited lexical decision tasks (Jacobs et al., 2003). In the case of words this allows for fast responses in response-limited lexical decision tasks. In the case of nonwords a temporal deadline mechanism was suggested to prolong processing times to allow for deeper inspection of the input resulting in longer response latencies for nonwords with many neighbors (Grainger and Jacobs, 1996).

So far, however, evidence of higher brain-electrical or hemodynamic activity for stimuli with many neighbors was inconclusive providing less support for a direct model-to-brain-data connection. Only two ERP studies reported results compatible with the idea of global lexical activity supporting lexical decisions. The first study found stronger N400’s in lexical and semantic decisions for stimuli with many neighbors and interpreted this activity as reflecting the sum of semantic activation of the target word and its neighbors (Holcomb et al., 2002). The second study found a parametric brain-electrical effect around 500 ms after stimulus presentation for nonwords differing in model-generated global lexical activity values that was interpreted to reflect the temporal deadline mechanism working differentially on nonwords with varying levels of orthographic similarity to the input (Braun et al., 2006). In contrast neuroimaging research rather showed contradicting evidence. One neuroimaging study showed a reversed effect of neighborhood density (i.e., higher activity for stimuli with a low number of neighbors) in language related areas such as the middle temporal and angular gyri suggested to reflect semantic processing (Binder et al., 2003). The other study found prefrontal activity in response to stimuli differing in number of neighbors which was interpreted as reflecting executive domain general processes related to post-lexical rather than lexical processing (Fiebach et al., 2007).

### Neighborhood Density Effect

The current study revealed for the first time a neighborhood density effect with higher activity for stimuli with many neighbors. Words and nonwords with many neighbors elicited greater BOLD responses in the dorsomedial prefrontal cortex (dmPFC) potentially signaling global lexical activity which is in support of models suggesting orthographic similarity as the basis of the neighborhood density effect. However, the results of previous neuroimaging studies make it rather unlikely that this dorsomedial prefrontal activity directly reflects activation of representations orthographically similar to the stimulus in a hypothetical mental lexicon. The dmPFC is known to be involved in higher order executive control processes like decision making, conflict monitoring, response conflict, theory of mind, and language comprehension (Bechara et al., 1998; Cohen et al., 2000a; Ferstl and von Cramon, 2002; Ridderinkhof et al., 2004; Rogers et al., 2004; Schurz et al., 2014b). Furthermore, there is much evidence pointing to a prominent role of the dorsomedial and ventrolateral prefrontal cortex in working memory (e.g., Rao et al., 1997; Henson et al., 1999). The functions of a hypothetical mental lexicon are rather associated with inferior parietal, middle and inferior temporal regions (e.g., Howard et al., 1992; Beauregard et al., 1997; Hagoort et al., 1999; Fiebach et al., 2002; Binder et al., 2003, 2009; Kronbichler et al., 2004). The results of the exploratory ROI analysis in the ventral occipitotemporal cortex supports this hypothesis. The neighborhood density effect near the coordinates of the VWFA (-42 -57 -15) identified by Cohen et al. (2002) for words and nonwords provides evidence that orthographic similarity is also differentially processed in this region for our items.

Since subjects in the current study only silently read the words and nonwords and no overt decisions had to be made, the observed dorsomedial prefrontal activity in our study is not likely to reflect decision-related processes. Rather, it seems that words and nonwords with many neighbors activate orthographically similar representations which elicit higher activity for these items in the dmPFC. We therefore suggest that this activation reflects the activation, maintenance, and monitoring of those representations orthographically similar to the presented stimuli, i.e., an implicit verbal working memory function.

Evidence for such a memory function in the dmPFC was reported by Henson et al. (1999) who reported higher activity for know-answers compared to remember-answers in an old-new paradigm with five-letter nouns in lexical decision. A remember answer was given in the case of surely remembered items seen before, a know-answer was given when subjects knew that the items were presented during the study phase, but could not recollect any contextual information about its previous occurrence. The higher activity for familiarity based judgments compared to surely identified items was proposed to reflect stronger monitoring demands when memory judgments are less certain. Furthermore, their results suggested a dissociation between activity in parietal and prefrontal areas: in contrast to the prefrontal activity in response to know answers surely remembered items elicited higher activity in parietal/temporal areas suggesting a differential processing for remember/know items. Henson et al. (1999) therefore proposed that surely remembered items are likely to be identified in parietal/temporal areas and that familiar items are processed in dmPFC (Miller and Cohen, 2001).

### Lexicality Effect

The assumption of lexico-semantic processing in the parieto-temporal region is in line with the obtained lexicality effect revealed by the whole-brain analysis in this region with higher activity for words irrespective of neighborhood density at the border of left angular, middle, and inferior temporal gyrus. We propose that this reflects lexico-semantic processing (Binder et al., 2003, 2009; Gold et al., 2005; Lau et al., 2008) based on local lexical activity as proposed by the MROM or the DRC (Jacobs and Grainger, 1994; Grainger and Jacobs, 1996; Coltheart et al., 2001). In the case of words this results in successful activation of a stored lexico-semantic representation whereas in the case of nonwords no such representation can be found resulting in greater deactivation of this region. This interpretation is also supported by the additional ROI analysis which revealed a main effect of lexicality but no main effect of neighborhood density and no interaction in the angular and middle temporal gyrus. We therefore suggest that the results of Henson et al. (1999) and the present lexicality effect in the inferior parietal and middle temporal cortex reflect the activation and retrieval of word-specific lexico-semantic information leading to higher activity for words compared to nonwords (cf. Binder et al., 2003). The obtained lexicality effect in the precentral and opercular cortex with higher activity for nonwords compared to words is probably related to the stronger demands on orthographic-phonological mapping during reading of nonwords (e.g., Bitan et al., 2007; Braun et al., 2009, 2015).

### Lexicality by Neighborhood Density Interaction

Furthermore, the current study revealed an interaction of lexicality by neighborhood density in left ventromedial prefrontal cortex. Nonwords with a high number of neighbors showed higher activity than words with a high number of neighbors in this region. This activity mirrors the BOLD response pattern obtained in lexical decision from Fiebach et al. (2007) in the medial superior frontal gyrus who suggested that it reflects extra-lexical processing in the form of a domain-general neural mechanism of executive control due to the processing demands of speeded lexical decisions. Fiebach et al. (2007) further suggested that the lower activity for words and the higher activity for nonwords with a high number of neighbors reflect executive control processes suppressing the global lexical activation elicited by the many neighbors of the target nonword. While this seems plausible for lexical decisions, it is rather unlikely for silent reading. Therefore, we would like to propose that the lexicality by neighborhood interaction is not due to response suppression or inhibition but rather to memory related processing in the ventromedial prefrontal cortex.

Recently, Harand et al. (2012) reported a number of prefrontal regions to be active during the remember/know task that are relevant to our interpretation of the neighborhood density effect in the prefrontal cortex. In particular, it was suggested that frontal nodes in this network subserves top–down attentional processes, involving inhibition, monitoring, and working memory operative in memory retrieval. Analogously, assuming that silent reading imposes only low demands on executive control processes the present activity in the ventromedial prefrontal cortex for words and nonwords with a high numbers of neighbors may reflect processes of working memory including maintenance, monitoring, and the verification of activated memory representations associated with presented targets.

Such an interpretation is also supported by studies investigating autobiographical, episodic, emotional, and semantic memory processes (Gilboa, 2004; Kuchinke et al., 2006; Burianova and Grady, 2007). Gilboa (2004) reported greater activity in the left ventromedial prefrontal cortex for autobiographical memory compared to episodic memory and suggested that remembering of autobiographical memories more strongly involves the monitoring of the accuracy and cohesiveness of retrieved memories in relation to an activated self-schema relying on a quick intuitive “feeling of rightness” (Elliott et al., 2000; Moscovitch and Winocur, 2002).

Moscovitch and Winocur (2002) introduced the term “felt rightness” to describe a possible role of the ventromedial prefrontal cortex in working memory. Felt rightness should refer to the ability to intuitively guess the correctness or accuracy of a response in relation to the goals of a memory task. Furthermore, these authors suggested that this kind of processing precedes an elaborate cognitive verification of the truthfulness of the memory and the context in which it is retrieved.

However, activity of the ventromedial prefrontal cortex is not restricted to autobiographical memory, but is also found in situations where responses are made by guessing under conditions of uncertainty (Nathaniel-James et al., 1997; Elliott et al., 2000). For example, Nathaniel-James et al. (1997) applied the hayling test (Burgess and Shallice, 1997) and found greater activation in the anterior ventromedial prefrontal cortex when participants had to complete sentences that had many possible correct completions compared to conditions with only a few possible correct completions.

Elliott et al. (2000) emphasized the role of the medial orbitofrontal cortex in monitoring and in “holding things in mind” and that this function applies especially to aspects of familiarity and rightness. They suggested that the ventromedial prefrontal cortex is more active in a working memory matching condition than in a non-matching condition which does not involve any guessing. In two tasks subjects were initially shown a complex, abstract visual stimulus, then after a delay interval, were confronted with two stimuli, one of which was the sample stimulus. In the delayed matching to sample, the subjects’ task was to choose the familiar stimulus; in the delayed non-matching task subjects had to choose the novel stimulus. When both conditions were compared, greater activation in the medial caudate and ventromedial orbitofrontal cortex was seen for the matching condition.

However, according to Elliott et al. (2000) the medial orbitofrontal activation in their matching task is unlikely to reflect working memory processes per se. They argued that in the matching condition an association between a specific stimulus and a forthcoming response can be formed and maintained through the delay interval. For the non-matching condition a sample stimulus does not specify a forthcoming response and therefore no association is formed. Therefore, the differential orbitofrontal activation may reflect the maintenance of stimulus–response mappings in the matching-to-sample task.

Since, in our silent reading study no stimulus–response mapping was required, the activity in the ventromedial prefrontal cortex is thus not likely to be related to stimulus–response mappings. We rather suggest that the lexicality by neighborhood density interaction observed in the ventromedial prefrontal cortex is mainly associated with the comparison/matching of the stimuli to stored representations orthographically similar to them.

We further propose that the obtained interaction in the ventromedial prefrontal cortex for words and nonwords with a high number of neighbors is not independent of the activity in the ventral occipitotemporal cortex (e.g., Cavada et al., 2000; Bar, 2003; Uylings et al., 2003) and to reflect the operation of different memory related processes. Words with a high number of neighbors elicited lower activity in the ventromedial prefrontal cortex compared to nonwords with a high number of neighbors. It is likely that words with many neighbors are fast and easily identified based on the processing in the VWFA eliciting a quick intuitive feeling of rightness which allows for a true fast-guess response in lexical decisions. In contrast, nonwords with a high number of neighbors elicited higher activity in the ventral occipitotemporal and ventromedial prefrontal cortex compared to words. This probably reflects more difficult matching processes in the VWFA and probably eliciting only a lower degree of felt rightness in the ventromedial prefrontal cortex which could lead to prolonged processing by extending a response deadline in lexical decisions for these stimuli.

The exploratory ROI analysis in the ventral occipitotemporal cortex with the main effects of lexicality and neighborhood density with lower activity for words compared to nonwords and lower activity for words and nonwords with many neighbors compared to those with few neighbors could support this view. The VWFA seems to be involved in the coding of the lexical status as well as the orthographic similarity of presented letter strings (Glezer et al., 2009; Baeck et al., 2015). The observed pattern of activity could reflect easier access to words analogously to the interpretation of lower activity in response to high frequency words compared to low frequency words in the VWFA by Kronbichler et al. (2004). Kronbichler et al. (2004) suggested that high frequency words and orthographically familiar forms allow for a fast assimilation of the letter input by readily available orthographic representations of specific words in the VWFA. It seems that the VWFA responds with lower activity to targets which are similar to known and already stored items compared to those which are less known or new (Kronbichler et al., 2004). This is also consistent with the finding of Glezer et al. (2015) who showed that word learning selectively increases neuronal specificity for new words in the VWFA which could point to the sensitivity of the VWFA’s in the processing of letter strings dependent on experience. In the light of these and our findings, we propose that words and nonwords with many neighbors are identified based on their orthographic similarity to whole-word representations in the VWFA which is further evidence for making this region a likely candidate for being part of an orthographic lexicon.

Concerning the model-to-brain-data connection it seems that there is no simple mapping between model activation and the hemodynamic activity in the VWFA or the ventromedial prefrontal cortex, as speculated by Jacobs and Carr (1995). Words with many neighbors produce high values of global lexical activity in the models but appear to elicit low hemodynamic activity. One possible explanation for this discrepancy is that the longer a stimulus is processed the higher the BOLD response (Buxton et al., 2004), an effect that was implemented in the ACT-R, for example (Anderson et al., 2004). Words and nonwords with a high number of neighbors may initially elicit a higher BOLD response because the orthography of these stimuli and their neighbors is more familiar and better represented and thus easier accessed. This leads to a faster identification and earlier termination of processing. In contrast, words and nonwords with a low number of neighbors activate fewer similar mental codes which probably also have a reduced or noisier orthographic representation and thus an initially lower BOLD response. This would make identification of these items more difficult and effortful as proposed by Taylor et al. (2012) in their engagement and effort model-to-brain-data connection hypothesis. The result would be prolonged processing and, in turn, by summation of activity over time, a higher BOLD response for words or nonwords with few neighbors. This explanation fits with simulations of the DRC, predicting that words with many neighbors need less processing cycles to reach an identification threshold, at least for low frequency words in lexical decision (Coltheart et al., 2001), compared to those with few neighbors (see Hofmann and Jacobs, 2014 for a similar explanation of word frequency effects).

## Conclusion

In sum, the present study sheds light on the neural bases of orthographic processing by investigating the neighborhood density effect in relation to the predictions of computational models of visual word recognition. We interpret the obtained activity in the dmPFC to mainly reflect processes of verbal working memory. This activity is modulated by the orthographic similarity of the presented words and nonwords to stored representations. We further suggest that the observed pattern of brain activity could reflect the operation of three mechanisms proposed by the above-mentioned models: (i) a fast-guess mechanism (Jacobs et al., 2003) based on the fast and easy visual identification of stimuli in the VWFA and on a spontaneous intuitive feeling of rightness of words with a high number of neighbors in the ventromedial prefrontal cortex; (ii) a deadline mechanism in the ventromedial prefrontal cortex which is supposed to prolong processing time for words with few and nonwords with many neighbors eliciting only lower levels of felt rightness. Thus, similar to the suggestion of Moscovitch and Winocur (2002) we think that the ventromedial prefrontal cortex may be involved in criterion setting for accepting or rejecting a memory trace. Both proposed mechanisms probably are at work during this process. In the case of words the fast-guess mechanism sets a positive criterion leading to fast identification and subsequent termination of processing. In the case of nonwords the temporal deadline mechanism sets a negative criterion which prolongs processing time for accepting or rejecting an item. In lexical decision the operation of both mechanisms results in shorter response latencies for words and longer response latencies for nonwords which probably is the basis of the dissociation of the neighborhood density effects previously found for words and nonwords with many neighbors. Finally, we propose the operation of an identification mechanism indicated by the lexicality effect in the inferior parietal and middle temporal cortex with higher activity for words compared to nonwords and propose that this activity reflects the identification of single items and their meaning based on local lexico-semantic activity.

## Conflict of Interest Statement

The authors declare that the research was conducted in the absence of any commercial or financial relationships that could be construed as a potential conflict of interest.

## Footnotes

1 http://www.neurobs.com/

2 http://www.fil.ion.ucl.ac.uk/spm/

3 http://dbm.neuro.uni-jena.de/vbm8
